# Supplementary material for: Structure of an RNA G-quadruplex from the West Nile virus genome
Source: Nat Commun. 2024 Jun 26;15:5428. doi: 10.1038/s41467-024-49761-5 (PMC11208454; doi:10.1038/s41467-024-49761-5)
Supplement: Supplementary file 1 — Supplementary Information [file 41467_2024_49761_MOESM1_ESM.pdf]

Structure of an RNA G-Quadruplex from the West Nile Virus Genome

Supplementary Tables and Figures

Table S1. WNV NS5-B WT and M1 RNA G4s and nucleic acids controls.[1]

	Name of sequence	Sequence	Length	Location on WNV NY99 genome
Controls	T_{20}	5'-TTT TTT TTT TTT TTT TTT TT -3'	20	
	RNA Duplex (dsRNA)	5'-CUG ACG AAG GCC UUC GUC AG-3'	20	
	c-MYC	5'-TGA **GGG** T **GGG** TA **GGG** T **GGG** TAA -3'	22	
	HTR	5'-**GGG** TTA **GGG** TTA **GGG** TTA **GGG**-3'	21	
WNV G4s	WNV-NS5-B WT	5'-UGU **GG** AAGA **GG** C **GG** UU **GG** UGU-3'	21	7925-7946
	WNV-NS5-B M1	5'-UAU **GG** AAGA **GG** C **GG** UU **GG** UAU-3'	21	
	WNV-NS5-B WT-27mer	5'-UGU **GG** AAGA **GG** C **GG** UU **GG** UGU UAC UAU-3'	27	7925-7952
	WNV-NS5-B WT-30mer	5'-UU **GG** AUGU **GG** AAGA **GG** C **GG** UU **GG** UGU UAC U-3'	30	7920-7950

[1]G runs are **bolded**; NS5-B sequences are blue; black are control sequences; red indicates nucleotide modifications.

Table S2. Selected base parameters of the NS5-B M1 structure[1].

`

Chain		1	2	3	4	5	6	7	8	9	10	11	12	13	14	15	16	17	18	19	20	21
A	NS5-B M1	U	A	U	G	G	A	A	G	A	G	G	C	G	G	U	U	G	G	U	A	U
	Base Orientation	anti	anti	anti	anti	anti	anti	anti	anti		anti	anti	syn	anti	anti	anti		anti	anti	anti	anti	anti
	Sugar Pucker	N	N	N	S	N	S	N	S	S	S	S	N	N	N	S	S	N	N	N	S	S
	Psuedorotation	7.1	11.4	15.8	161.4	22.2	176.6	18.4	155.2	145.5	151.2	159.5	43.3	358.6	5.7	161.8	157.7	0.9	18.9	13.3	158.7	156.3
	χ	-175.9	-166.5	-150.4	-130.7	-144.3	-103.8	-141.1	-125.0	-22.9	-117.9	-115.4	91.9	-178.2	-159.3	-122.1	26.7	-164.3	-154.9	-151.1	-128.4	-119.7
B	Base Orientation	anti	anti	anti	anti	anti	anti	anti	anti		anti	anti	anti	anti	anti	anti	anti	anti	anti	anti	anti	anti
	Sugar Pucker	N	N	N	S	N	S	N	S	S	N	S	S	N	N	S	S	N	N	N	S	S
	Pseudorotation	8.7	6.5	11.3	161.8	22.3	174.1	14.7	157.6	147.2	44.2	158.2	118.9	358.6	7.2	159.7	157.5	11.3	17.4	14.4	161.7	155.8
	χ	-170.0	-167.8	-147.4	-126.3	-143.8	-98.7	-147.9	-121.0	-30.4	-171.4	-115.4	-117.7	-178.3	-158.3	-118.9	-126.9	-176.1	-156.4	-150.6	-131.9	-127.4

[1] Differences between Chain A & Chain B are highlighted in grey.

Table S3. Modified NS5-B M1 sequences.[1]

WNV	1	2	3	4	5	6	7	8	9	10	11	12	13	14	15	16	17	18	19	20	21
NS5-B WT	U	G	U	G	G	A	A	G	A	G	G	C	G	G	U	U	G	G	U	G	U
NS5-B M1	U	A	U	G	G	A	A	G	A	G	G	C	G	G	U	U	G	G	U	A	U
NS5-B M1 G8A	U	A	U	G	G	A	A	<u>A</u>	A	G	G	C	G	G	U	U	G	G	U	A	U
NS5-B M1 D2	U	A	U	G	G	A	A	G	A	G	G	C	G	C	U	U	G	G	U		
NS5-B M1 D3	U	A	U	G	G	A	A	G	A	G	G	C	G	C	U	U	G	G			

[1] Bases making up the α-tetrad are in teal, the β-tetrad bases are in green, the γ-triad bases are in orange and the dyad bases are in grey. Mutation to the sequence is underlined.

Table S4. Viral genome sequences used in the Figure 4 alignment.

Virus	NCBI Accession #
WNV (NY99)	MZ605381.2
WNV (EG101)	AF260968.1
WNV (A956)	NC_001563.2
DENV 1	NC_001477.1
DENV 2	NC_001474.2
DENV 3	NC_001475.2
DENV 4	NC_002640.1
JEV	NC_001437.1
MVE	NC_000943.1
POWV	NC_003687.1
TBEV	NC_001672.1
YFV	NC_002031.1
ZIKV (PRVABC59)	KU501215.1
ZIKV (MR766)	NC_012532.1

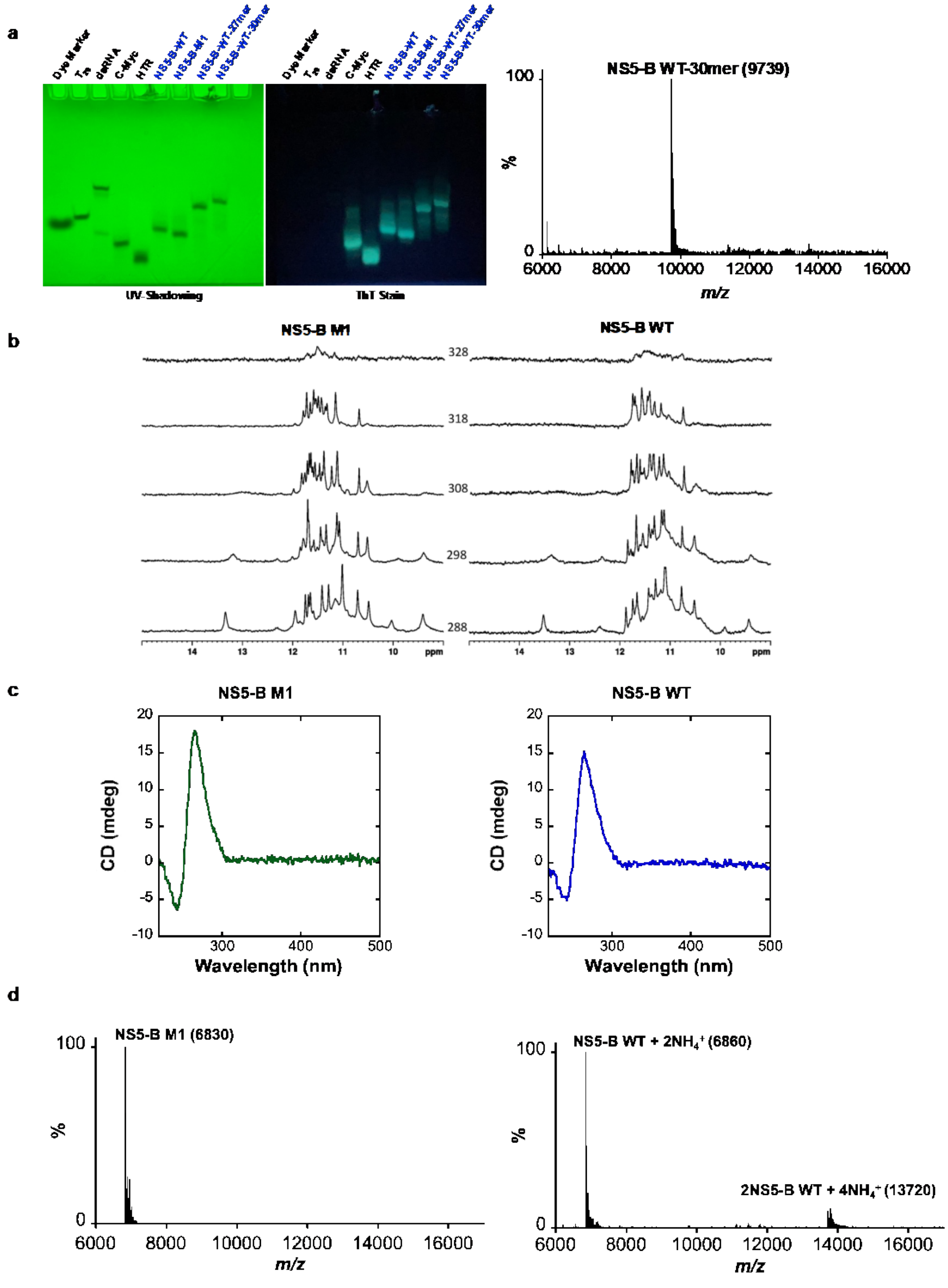

Figure S1. **Biophysical characterization of NS5-B wild-type and M1 mutant.** a) RNA migration of NS5-B wild-type and M1 sequences on Native PAGE detected by UV shadow and ThT stained (left). ESI-MS of the 30 nucleotides NS5-B wild-type sequence (right) indicates formation of monomeric quadruplex structure of ~10 kDa. b) 1D ^{1}H NMR spectrum of NS5-B M1 and WT recorded at different temperatures exhibit quadruplex characteristic imino proton signal at 10-12 ppm. c) CD spectrum of M1 and WT with characteristic parallel quadruplex signals at l_{max} = 264 nm and l_{min} = 245 nm. d) ESI-MS indicates both M1 and WT formed monomeric quadruplex structures of ~7 kDa. Source data are provided as a Supplemental Source Data file.

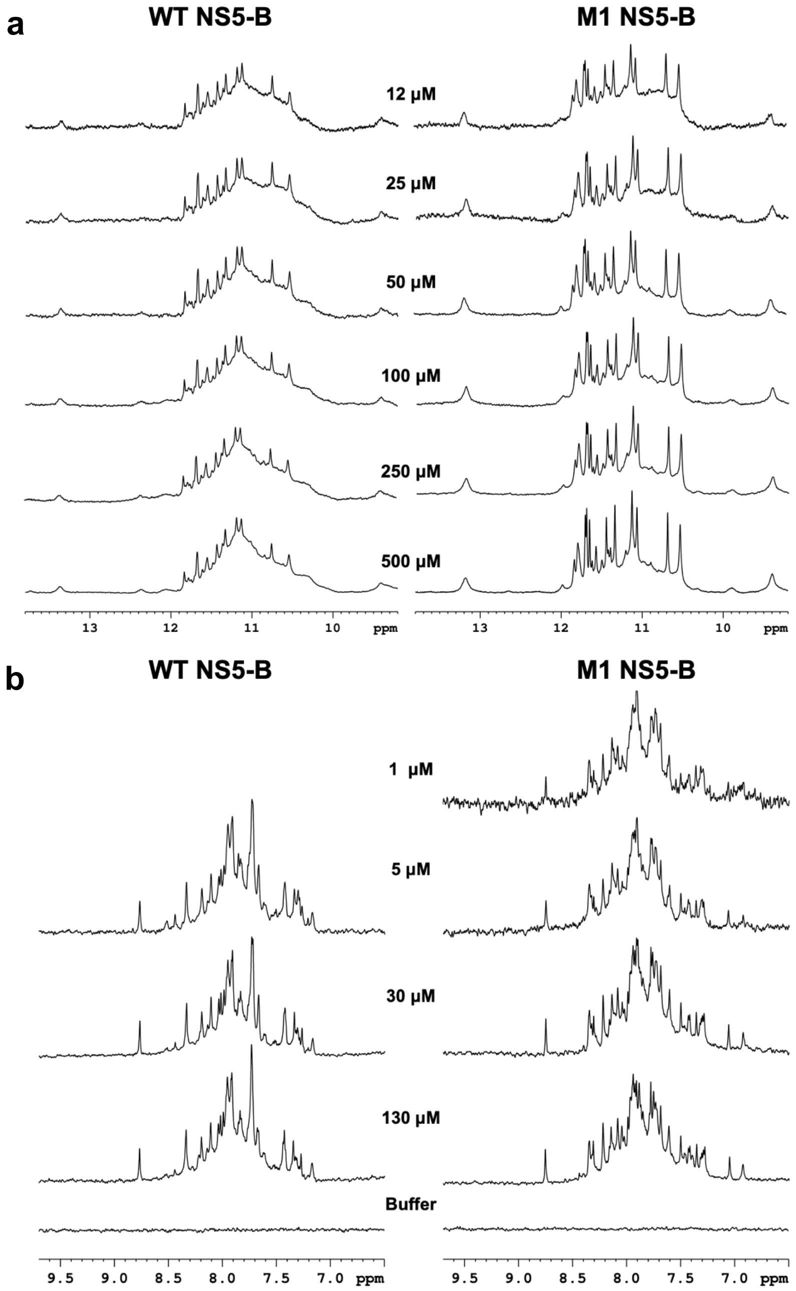

Figure S2. Concentration independent 1D ^{1}H NMR spectra of NS5-B WT and M1. ^{1}H NMR spectra were collected on a Bruker Avance 600 MHz NMR equipped with a 5 mm QXI probe. a) ^{1}H imino spectra were acquired using a modified jump and return pulse sequence at 297 K. RNA samples were diluted with NMR sample buffer containing 20 mM potassium phosphate, 50 mM KCl, 0.5 mM EDTA, and 10% D_2O at pH 6.5. Sample concentrations ranged from 500 μM to 12 μM. b) ^{1}H spectra of the aromatic region were acquired using a presat pulse sequence at 297 K. RNA samples were diluted with NMR sample buffer in 100% D_2O, pH* 6.4 with concentrations ranging from 130 μM to 5 μM for WT or to 1 μM for M1.

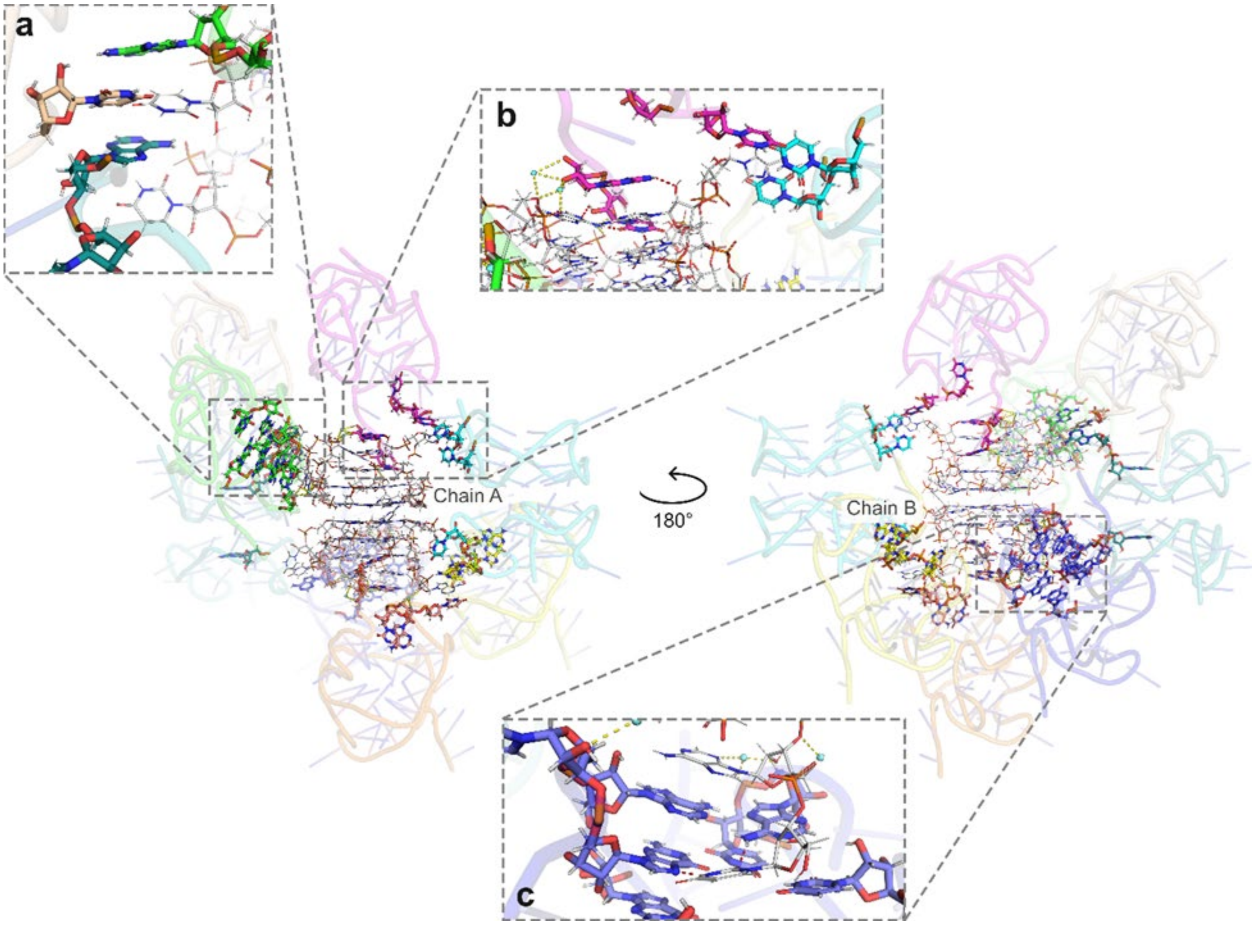

Figure S3: Crystallographic contacts in the NS5-B M1 quadruplex structure (8UTG). Shown are asymmetric units within 4.5 Å from the central NS5-B M1 quadruplex (rendered in thin sticks with white C atoms). The adjacent asymmetric units are rendered as cartoons in single colors. Ions and solvent atoms are omitted for clarity. Residues containing atoms within 4.5 Å from the central quadruplex are rendered as thicker sticks. Directional crystal contacts (i.e., H-bonds) between nucleic acids are rendered as red dashes, and water-mediated contacts as yellow dashes. Nucleotides denoted with * in the figure legend indicate a residue from an adjacent asymmetric unit. **Inset a:** Crystallographic contacts stabilize externally oriented conformers of chain A residues U1*, A2*, A7*, U16, and U21. **Inset b:** Contacts of triad/dyad chain A nucleotides with U1* and A2* from an adjacent asymmetric unit. **Inset c:** Contacts of triad/dyad chain B nucleotides with U1* and A2* from an adjacent asymmetric unit.

Supplemental Discussion of Crystal Contacts

Chain A to Chain B: Within the asymmetric unit, chains A and B primarily interact by π-stacking of the quadruplexes and by a direct hydrogen bond between G13(A) ribose-2′OH and a G4(B) phosphate oxygen. In addition, A9(A) and C12(B) form a single hydrogen bond, but this interaction appears to be dynamic per the high B-factors of the C12 from chain B. A9(B) and C12(A) form a π-stacking interaction which may serve to constrain the configuration of the C12(A).

Chain A external contacts: Inset a: Crystallographic contacts stabilize externally oriented conformers of residues U1*, A2*, A7*, U16, and U21 through π-stacking interactions. **Inset b:** U1* and A2* from an adjacent asymmetric unit project into the triad/dyad and may promote stabilization as a direct hydrogen bond forms between U1* and the N7 position of G8 in the triad as well as with the N10 position of A20 in the above dyad. A2* π-stacks above A6 and forms direct hydrogen bonds with the A6 2′ ribose-OH and the A20 ribose oxygen. Water-mediated backbone contacts between the A2* and A20 are also observed. The formation of the α- and β-tetrads do not appear to be externally stabilized as there are no crystal contacts occurring within 4.5 Å of chain A in these regions. Both A9 and C12 contain sufficient space for alternative conformations but retain their positions stabilizing the α-tetrad.

Chain B external contacts: Crystallographic contacts stabilize externally oriented conformers of residues U1, A2, A7, U16, and U21 via water-mediated H-bonds and π-stacking interactions. **Inset c:** In chain B, U1* and A2* from an adjacent asymmetric unit also project into the triad/dyad and may promote stabilization as a direct hydrogen bond that forms between U1* and the N7 position of G8 in the triad as well as with the N10 position of A20 in the above dyad. Once more, A2* π-stacks above A6 and forms direct hydrogen bonds with the A6 2′ribose-OH and the A20 ribose oxygen.

As with chain A, α- and β- tetrads are not externally stabilized as no crystal contacts occur within 4.5 Å of chain B in these regions. Both A9 and C12 contain sufficient space for alternative conformations and C12(B) is observed to be highly dynamic with elevated B-factors moving away from the phosphate backbone.

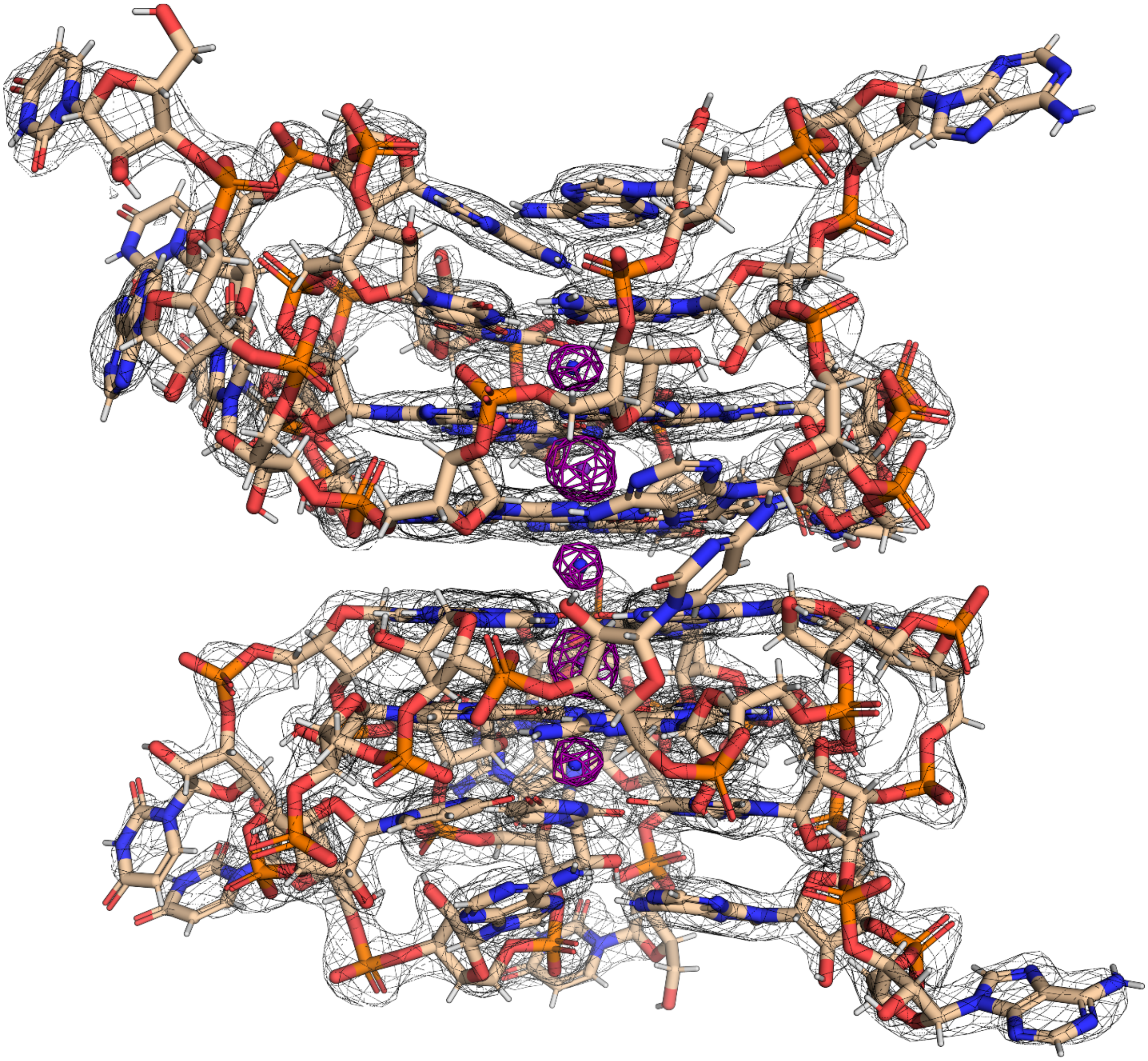

Figure S4. 2mFo-DFc difference map of the NS5 B M1 quadruplex contoured at 1.0 σ cutoff. Densities for the central cations are rendered in purple. K^+ is shown as purple and NH_4^+ as blue nonbonded spheres (without H atoms) to avoid obscuring the contours. Note the alternating sizes of the densities for K^+ and NH_4^+.

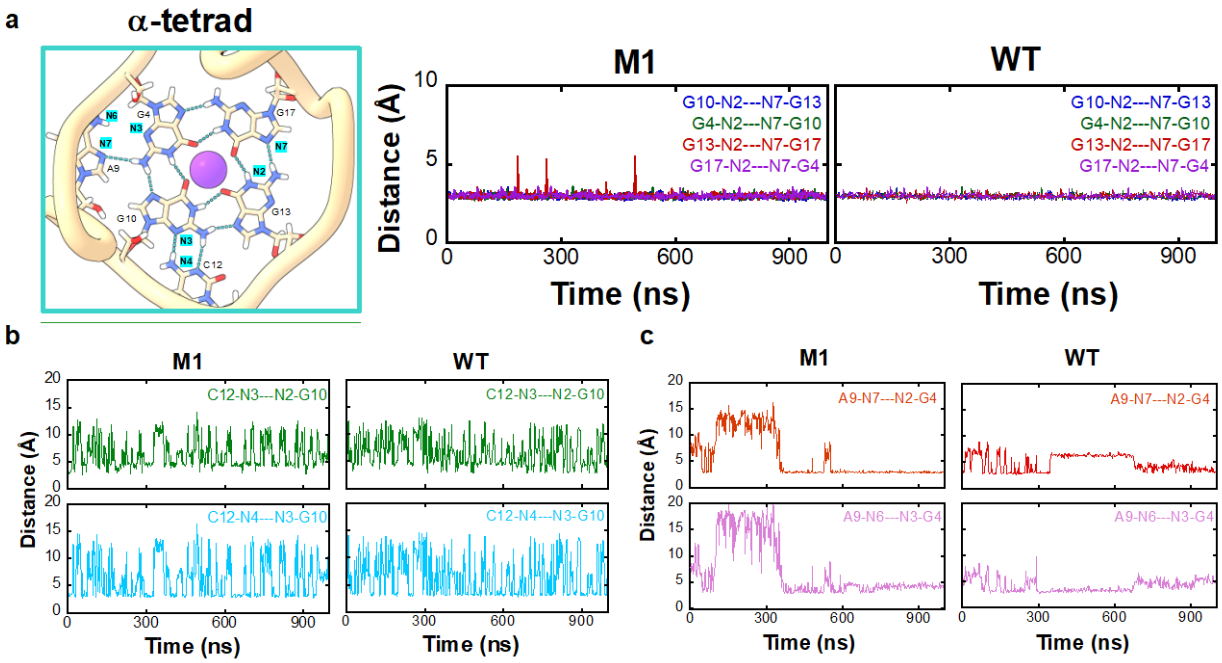

Figure S5. **MD analysis of the α-tetrad for NS5 B M1 and WT G-quadruplex structures.** A) The G17-G4-G10-G13 H-bond interactions at α-tetrad and the distance plots between the G17-G4-G10-G13 bases for M1 and WT. b) The distance plots between C12-G10 and c) between A9-G4. The time interval for each frame in the plot is 20 ps of a 1 μs trajectory (total 50,000 frames). Source data are provided as a Supplemental Source Data file.

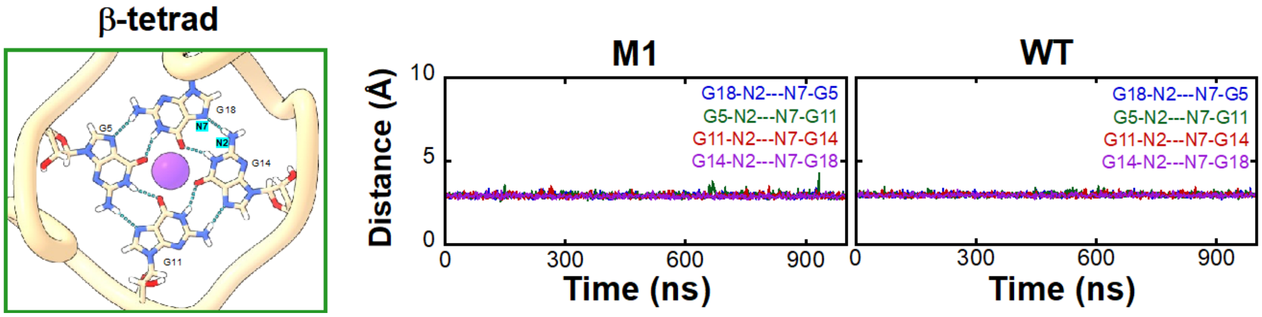

Figure S6. MD analysis of the β-tetrad for NS5-B M1 and WT G-quadruplex structures. a) The G18-G5-G11-G14 H-bond interactions at the β-tetrad and the distance plots between the G18-G5-G11-G14 bases for M1 and WT. The time interval for each frame in the plot is 20 ps of a 1 μs trajectory (total 50,000 frames). Source data are provided as a Supplemental Source Data file.

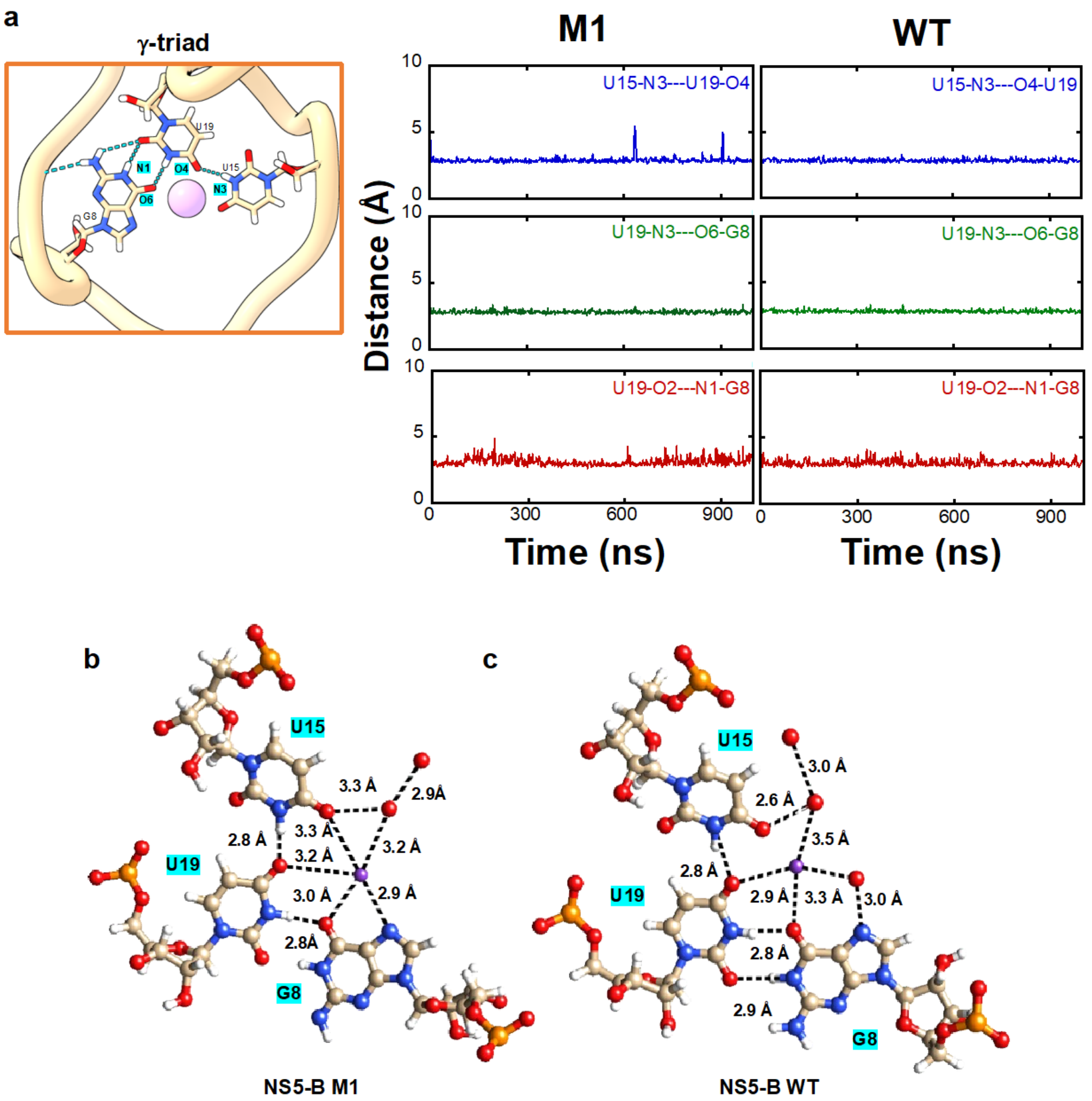

Figure S7. MD analysis of the γ-triad for the NS5-B M1 and WT G-quadruplex structures. a) The U15-U19-G8 H-bonding interactions at γ-triad for NS5-B M1 and the distance plots between the U15-U19-G8 bases for M1 and WT. The time interval for each frame in the plot is 20 ps of a 1 μs trajectory (a total of 50,000 frames). b) The snapshots of extended water network and K^+ ion interactions in the NS5-B M1 and c) NS5-B WT triad. The RNA bases are represented in a tan-white-blue-red (C-H-N-O) color scheme. The waters are represented in red, ball from ball & stick model, and the K^+ in purple, ball from ball & stick model. Source data are provided as a Supplemental Source Data file.

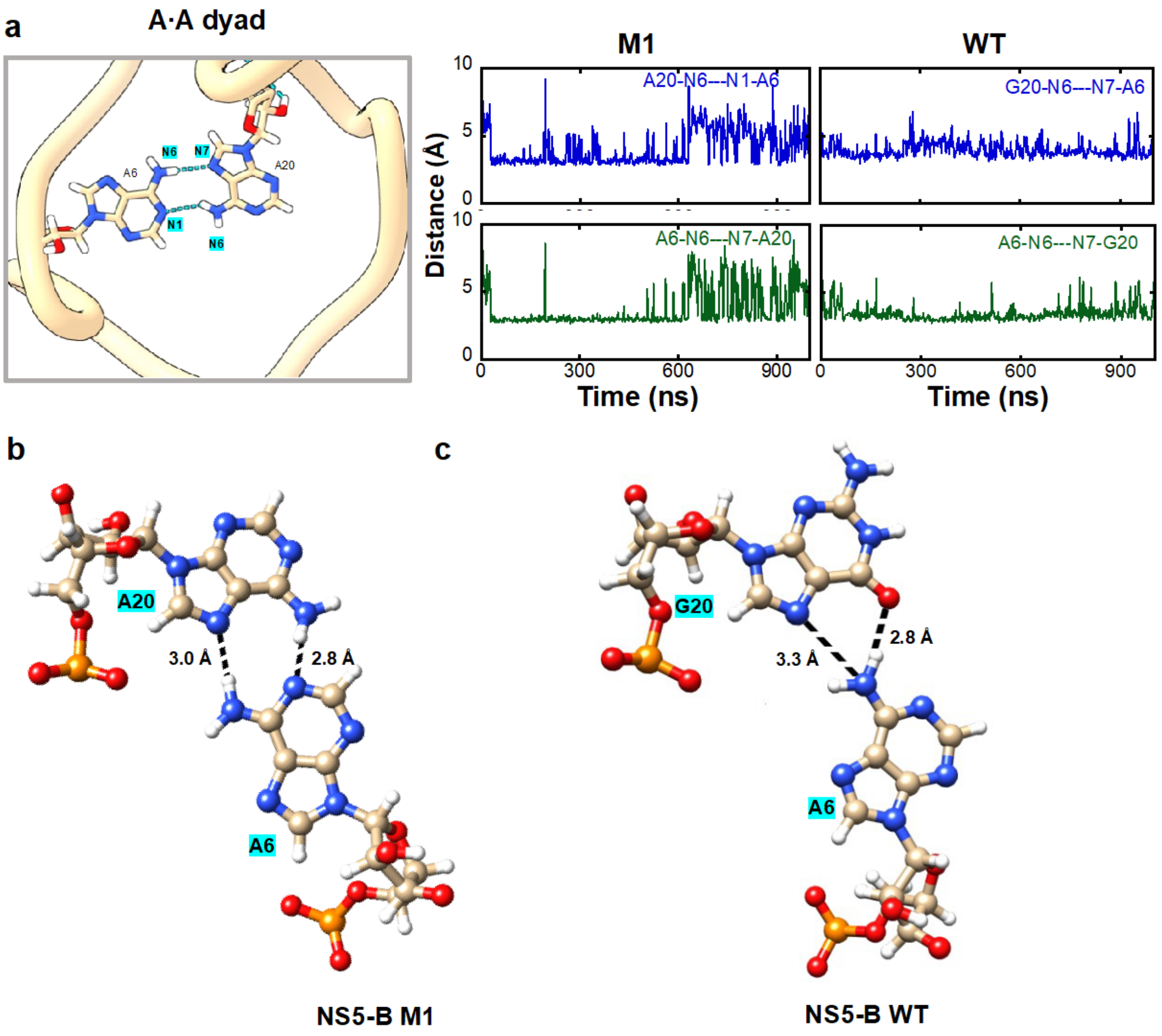

Figure S8. MD analysis of the dyad for the NS5-B M1 and WT G-quadruplex structures. a) The A20-A6 H-bonding interactions at dyad of NS5-B M1 and the distance plots between the RNA bases at the top cap base pair of A6 and A20 for M1 and A6 and G20 for WT. The time interval for each frame in the plot is 20 ps of a 1 µs trajectory (a total of 50,000 frames). MD snapshots of the H-bonding interactions of b) A20-A6 in NS5-B M1 and c) G20-A6 in NS5-B WT. The RNA bases are represented in a tan-white-blue-red (C-H-N-O) color scheme. Source data are provided as a Supplemental Source Data file.

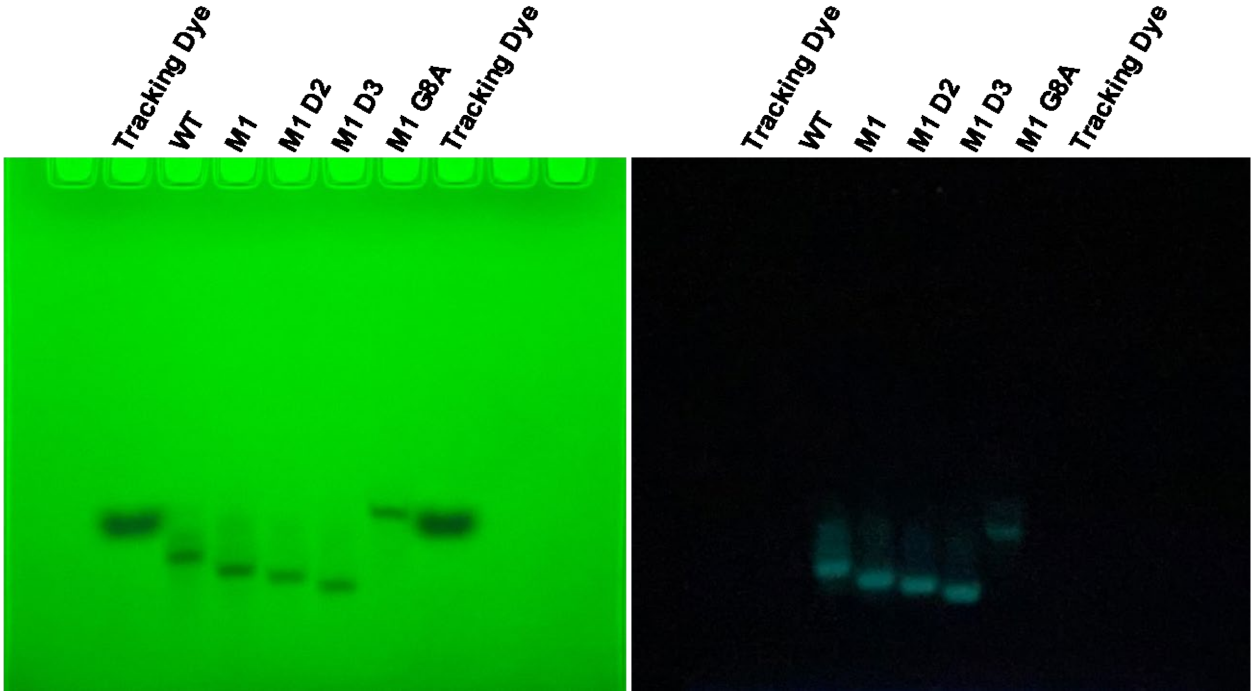

Figure S9. Native PAGE of NS5-B WT and M1 mutants. The migration of the RNA was detected by UV-shadowing (left) and ThT stain (right). On a 15% Native PAGE, all NS5-B sequences migrate faster or at the same rate as the bromophenol blue tracking dye which has a similar mobility to a 20-mer sequence; in addition, the ThT fluorescence enhancement, data indicate the sequences form monomeric quadruplex structures.

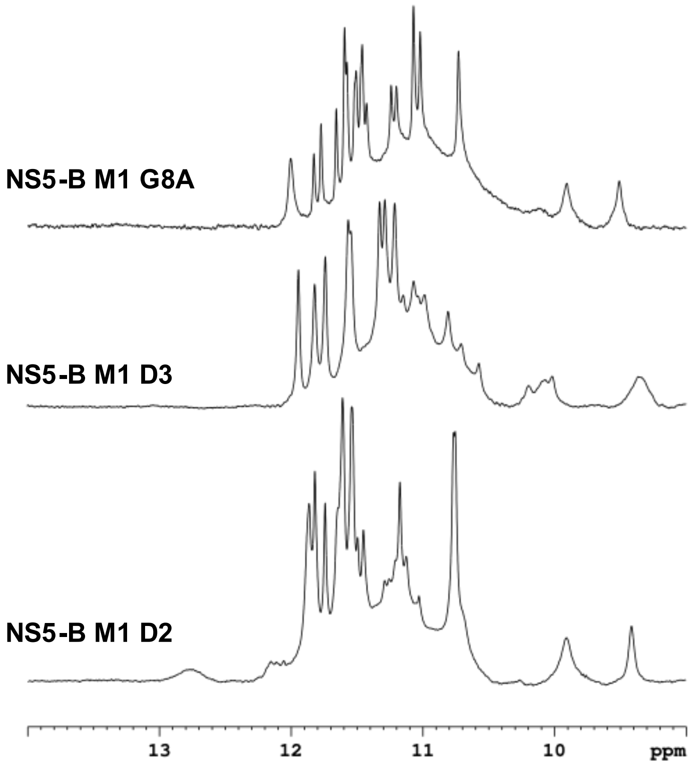

Figure S10. 1D ^{1}H NMR spectra of NS5-B M1 mutants. ^{1}H NMR spectra were collected on a Bruker Avance 600 MHz NMR equipped with a 5mm QXI probe. ^{1}H imino spectra were acquired using a modified jump and return pulse sequence at 288K. RNA samples were diluted with NMR sample buffer containing 20 mM potassium phosphate, 50 mM KCl, 0.5 mM EDTA, and 10% D_2O to achieve a final concentration of 100 µM. Samples were adjusted to pH 6.5. The NS5-B M1 D2 mutant which has two of the 3' terminal nucleotides deleted retains a signal around 12.8 ppm. However, both the D3 deletion of the U19-U21 and G8A mutation, which targets the triad, removes the imino proton signals above 12 ppm.

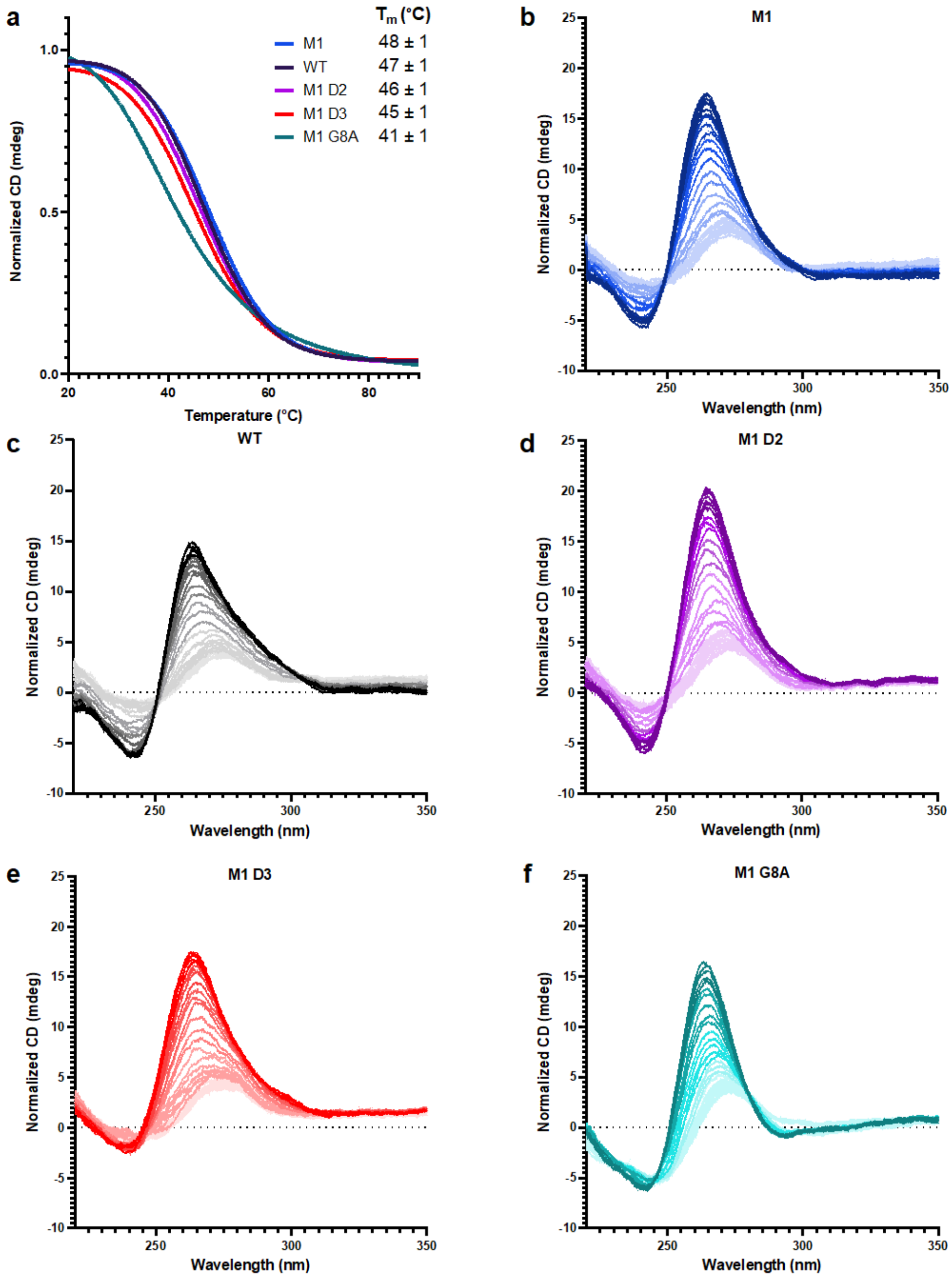

Figure S11. Thermal denaturing profile and CD spectra of NS5-B WT and M1 mutants. a) Normalized CD thermal denaturing curves and T_m values of WT and M1 mutants measured at 264 nm. b-f) Thermal denaturing CD spectra of WT and M1 mutants from 20°C to 90°C. RNA samples were prepared at 4 µM concentrations in 20 mM potassium phosphate, 50 mM KCl and 0.5 mM EDTA at pH 6.5. The T_m of M1 D2, M1 D3 and M1 G8A mutants (46°C, 45°C and 41°C) are lower compared to the T_m of M1 and WT (48°C, 47°C), indicating reduced G-quadruplex structure stability in the mutants. Source data are provided as a Supplemental Source Data file.

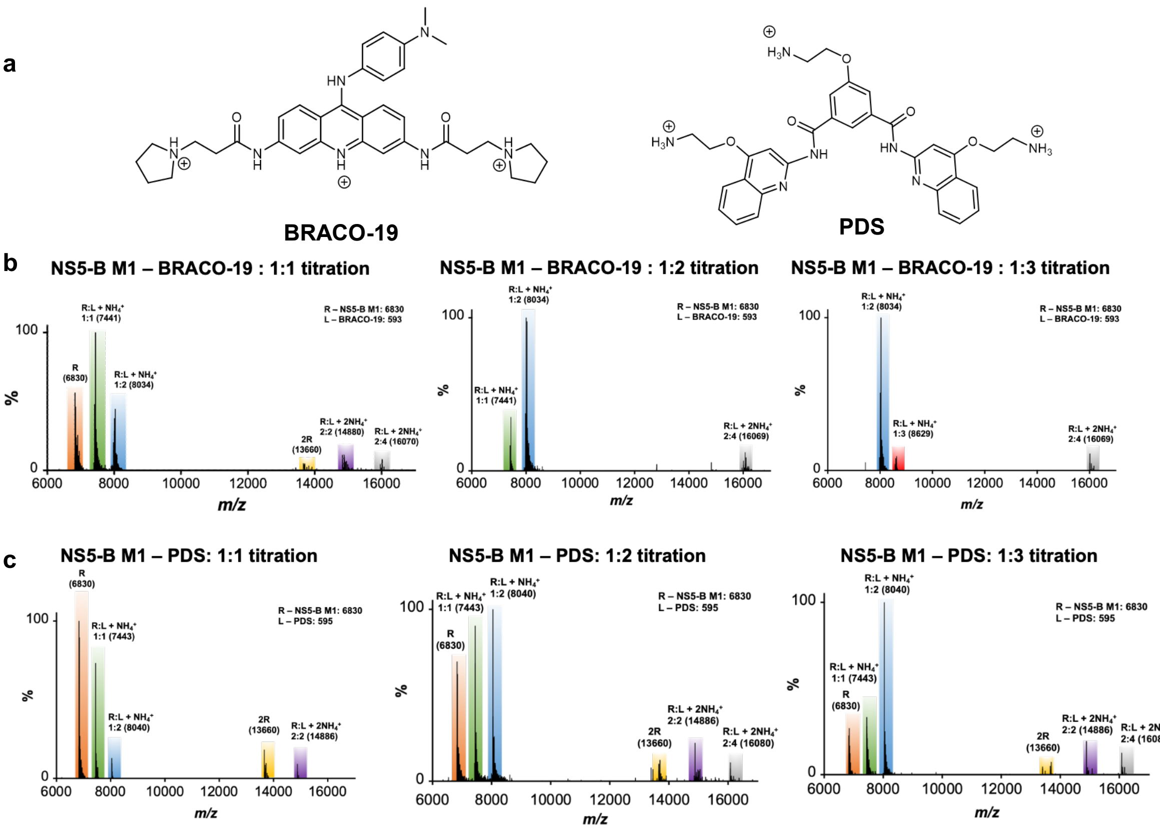

Figure S12. ESI-MS titration of commercially available G-quadruplex binders, BRACO-19 and PDS with NS5-B M1 RNA. a) Structure of BRACO-19 and PDS. b) Titrations of BRACO-19 to NS5-B M1 indicate BRACO-19 binds to NS5-B M1 at a 1:1 (7441), a 1:2 (8034) and a 1:3 ratio minor interaction (8629). c) Titrations of PDS to NS5-B M1 indicate PDS binds to NS5-B M1 at a 1:1 (7443) and a 1:2 ratio (8040). At higher molecular weights, for both BRACO-19 and PDS, the small peaks indicate association of two RNA G4s with ligands, respectively. Source data are provided as a Supplemental Source Data file.
