# Supplementary material for: Structure of an RNA G-quadruplex from the West Nile virus genome
Source: Nat Commun. 2024 Jun 26;15:5428. doi: 10.1038/s41467-024-49761-5 (PMC11208454; doi:10.1038/s41467-024-49761-5)
Supplement: Supplementary file 3 — Description of Additional Supplementary Files [file 41467_2024_49761_MOESM3_ESM.pdf]

<u>Description of Additional Supplementary Files</u>

Supplementary Movie 1: A video of the 8UTG structure rotated 360 degrees.
